# 

**Published:** 1906-09

**Authors:** 


					CONTENTS.
Past
The Adaptation of Means in the Prevention of Disease.
By
D. S. Davies, M.D.Lond., D.P.H.Cantab.
193
The Treatment of Tabetic Ataxia by Methodical Exercises.
By
Maurick Faure, M D.
^ Case of Mesenteric Thrombosis.
By G. Munro Smith, M.R.C.S.
216
On the Treatment of Diabetes with Secretin.
By J. R. Charles,
M.A., M.D.Cantab.. M.K.C.F.
218
The Doctor as Expert and Pioneer.
By Charles J. Whitby, M.D.
?"he Uses of Static Electricity as an Aid to Recovery.
By Preston
King. M.A.. M.D. Cantab.
233
'he Explanation of Alveolar Cleft Palate.
By Edward Fawcett,
M.D.Edin.
237
Progress of the Medical Sciences:
Medicine.
T. Michell Clarke, M.A., M.U.^antaD.
249
Surerery.
T. Carwardine, M.S., M.B.Lond., t.K.U.b.
254
? Ophthalmoloey.
Cyril H. Walker, M.B., M A.Cantab., F.R.C.S.
250
Reviews of Books
26i
Editorial Notes
268
Notes on Preparations for the Sick
281
[The Library of the Bristol Medico-Chirurgical Society   284
ybituarv Notice ...
286
i^ocal Medical Notes
287

				

